# The social defeat hypothesis of schizophrenia: a parsimonious explanation for multiple psychosis risk factors?

**DOI:** 10.1017/S0033291720004092

**Published:** 2023-01

**Authors:** Rik Schalbroeck

**Affiliations:** 1 Rivierduinen Institute for Mental Healthcare, Leiden, The Netherlands; 2 School for Mental Health and Neuroscience, Maastricht University, Maastricht, The Netherlands

**Keywords:** Migration, psychosis, schizophrenia, social defeat

In their recent meta-analysis, Selten, van der Ven, and Termorshuizen (2020) report an increased risk of psychotic disorder in first- and second-generation migrants, and show that this risk is associated with skin colour and region of origin and destination. The authors argue that the social defeat hypothesis of schizophrenia offers an explanation for these findings. However, this hypothesis has limitations related to the definition and measurement of social defeat, which hinder its use as a scientific explanation for the risks of psychosis in migrants and other groups.

The social defeat hypothesis states that the negative experience of being excluded from the majority group (i.e. social exclusion) leads to sensitization and/or increased baseline activity of the mesolimbic dopamine system and thereby to an increased risk of schizophrenia or psychosis (Selten, van der Ven, Rutten, & Cantor-Graae, 2013). The hypothesis originally aimed to provide a parsimonious explanation for four psychosis risk factors, including migration, low intelligence quotient (IQ), illicit drug use, and urban upbringing (Selten & Cantor-Graae, 2005), and was later also applied to groups such as individuals with autism spectrum disorder (ASD) and lesbian, gay, and bisexual individuals.

At first glance, it might seem defensible that social defeat is a common risk factor between these groups. However, since social defeat has been defined inconsistently, it is difficult to evaluate whether it truly is associated with the risk of psychosis in these individuals. That is, social defeat is defined in some publications (including the present meta-analysis) as social exclusion (e.g. Selten et al., 2013, 2020), and in other publications as subordinate position or outsider status (e.g. Selten, Booij, Buwalda, & Meyer-Lindenberg, 2017; Selten & Cantor-Graae, 2005). Although these constructs can be related, their interchangeable use is unjustified since they are not equivalent (e.g. in contrast, a person can be in a subordinate position within a majority group).

As a result, it is unclear whether the hypothesis proposes that subordinate position, outsider status, social exclusion, or some combination of them accounts for the observed risks of psychosis in migrants and other groups (this issue is complicated even further when definitions from animal and depression research are taken into account, which define defeat in terms of losing and/or a sense of failed struggle; Gilbert, Allan, Brough, Melley, and Miles, 2002). This lack of uniform definition of defeat also demonstrably results in conflicting interpretations of the hypothesis. For example, social defeat was operationalized in one study as loneliness as this resembled the subjective experience of social exclusion (Gevonden et al., 2014), and in another as low social rank, which was contrasted with loneliness (Jaya, Ascone, & Lincoln, 2017).

A second limitation of the hypothesis is that the authors argue that defeat can be difficult to measure because people do not want to admit having this experience (e.g. Selten et al., 2013). This statement is problematic for several reasons. First, it is unclear what people do not want to admit since social defeat has not been defined uniformly. Second, one can question whether this statement is true, as other studies did, for instance, measure experiences of outsider status and reduced value (van Nierop et al., 2014). Third, this statement limits the hypothesis' falsifiability, as it provides researchers with an excuse in the case of null-findings, allowing them to claim that they found no relationship between reports of social defeat and psychosis because their subjects did not want to admit feeling defeated.

Several solutions to the problem of immeasurability have been proposed. First, the authors state that the hypothesis can be tested by examining risks of psychosis and/or dopaminergic functioning in groups presumed to be socially defeated (Selten et al., 2013, 2020). However, often it is nearly impossible to unequivocally establish whether (or to what extent) a group is socially defeated, since the experience of defeat is subjective and does not necessarily vary in proportion to exposure to social adversity (Selten & Cantor-Graae, 2005). For example, one researcher might argue that individuals with ASD experience more social adversity and thus feel more defeated, whereas another might argue the opposite by stating that these individuals do not care about or notice their social adversity. Subsequent empirical observations then still cannot test the hypothesis. Moreover, groups presumed to be socially defeated usually share exposure to other factors, such as threat and negative affect, which could alternatively underlie observed risks of psychosis.

Second, it has been suggested that alternative measurements (e.g. of certain cognitive schemas or self-esteem) can be used to (indirectly) assess social defeat (Selten et al., 2013). However, these measurements (and/or complex interactions between them) might be associated with the development of psychosis even if they are unrelated to social defeat, and such unique contributions are likely underestimated or missed if they are solely interpreted as being indicative of social defeat.

In the past 15 years the social defeat hypothesis of schizophrenia has provided an intuitive and influential explanation for the role of the social environment in the development of psychosis. It has had demonstrable predictive power and seems to grasp some aspect of the social world that is important for understanding the development of psychosis. However, its use as a scientific hypothesis is hindered by the unclear definition and presumed immeasurability of social defeat. As a result, at this time it cannot be convincingly argued that the social defeat hypothesis provides a parsimonious explanation for the risk of psychosis in migrants and other groups.
